# The apoptotic effect of 1’S-1’-Acetoxychavicol Acetate (ACA) enhanced by inhibition of non-canonical autophagy in human non-small cell lung cancer cells

**DOI:** 10.1371/journal.pone.0171329

**Published:** 2017-02-03

**Authors:** Sophia P. M. Sok, Norhafiza M. Arshad, Mohamad Nurul Azmi, Khalijah Awang, Bulent Ozpolat, Noor Hasima Nagoor

**Affiliations:** 1 Institute of Biological Sciences (Genetics and Molecular Biology), Faculty of Science, University of Malaya, Kuala Lumpur, Malaysia; 2 Centre for Research in Biotechnology for Agriculture (CEBAR), University of Malaya, Kuala Lumpur, Malaysia; 3 Centre of Natural Product Research and Drug Discovery (CENAR), Department of Chemistry, Faculty of Science, University of Malaya, Kuala Lumpur, Malaysia; 4 Department of Experimental Therapeutics, The University of Texas MD Anderson Cancer Center, Houston, Texas, United States of America; National Institute of technology Rourkela, INDIA

## Abstract

Autophagy plays a role in deciding the fate of cells by inducing either survival or death. 1’S-1-acetoxychavicol acetate (ACA) is a phenylpropanoid isolated from rhizomes of *Alpinia conchigera* and has been reported previously on its apoptotic effects on various cancers. However, the effect of ACA on autophagy remains ambiguous. The aims of this study were to investigate the autophagy-inducing ability of ACA in human non-small cell lung cancer (NSCLC), and to determine its role as pro-survival or pro-death mechanism. Cell viability assay was conducted using MTT. The effect of autophagy was assessed by acridine orange staining, GFP-LC3 punctate formation assay, and protein level were analysed using western blot. Annexin V-FITC/PI staining was performed to detect percentage of cells undergoing apoptosis by using flow cytometry. ACA inhibits the cell viability and induced formation of cytoplasmic vacuoles in NSCLC cells. Acidic vesicular organelles and GFP-LC3 punctate formation were increased in response to ACA exposure in A549 and SK-LU-1 cell lines; implying occurrence of autophagy. In western blot, accumulation of LC3-II accompanied by degradation of p62 was observed, which further confirmed the full flux of autophagy induction by ACA. The reduction of Beclin-1 upon ACA treatment indicated the Beclin-1-independent autophagy pathway. An early autophagy inhibitor, 3-methyaldenine (3-MA), failed to suppress the autophagy triggered by ACA; validating the existence of Beclin-1-independent autophagy. Silencing of LC3-II using short interfering RNA (siRNA) abolished the autophagy effects, enhancing the cytotoxicity of ACA through apoptosis. This proposed ACA triggered a pro-survival autophagy in NSCLC cells. Consistently, co-treatment with lysosomal inhibitor, chloroquine (CQ), exerted a synergistic effect resulting in apoptosis. Our findings suggested ACA induced pro-survival autophagy through Beclin-1-independent pathway in NSCLC. Hence, targeting autophagy pathway using autophagy inhibitor such as CQ represented a novel promising approach to potentiate the cytotoxicity of ACA through apoptosis in NSCLC.

## Introduction

Lung cancer is the most common cancer worldwide; accounting for 1.82 million new cases and 1.6 million deaths in 2012 [1]. Among the lung cancer cases, non-small cell lung cancer (NSCLC) contributes to approximately 85% and has a low 5-year survival rate [2]. Conventional cancer therapies such as surgery, chemotherapy and radiotherapy were found to have limitation in maintaining its effectiveness during the course of therapy which lead to recurrence and acquired apoptosis resistance in long term treatment [3]. Hence, it is crucial to elucidate the underlying reason to improve the efficiency of the available therapeutic agents. Emerging evidences proposed that identifying the role played by autophagy in cancer could be a strategy to overcome resistance towards chemotherapy due to the fact its potential in eliciting a pro-survival or pro-death effect in response to metabolic and therapeutic stresses [4, 5].

Autophagy is a self-eating mechanism that is highly regulated by a set of autophagy-related (*atg*) genes to maintain the homeostasis in the cells through recycling of cellular components upon lysosomal degradation [6, 7]. Unlike canonical autophagy, a non-canonical autophagy occurs when certain ATG proteins such as ULK1, Beclin-1, class III phosphotidylinositol-3-kinase (PI3K) and Atg conjugation system are being bypassed during initiation of autophagy and formation of autophagosome [6, 7]. It was believed that autophagy induction often occur prior apoptosis to provide energy to allow survival of cancerous cells in response to stress such as nutrient deprivation or chemotherapy stress [8, 9]. Conversely, natural bioactive compounds such as silvestrol and guttiferon K were proven to elicit autophagy that lead to cell death [10, 11]. To date, the role of autophagy in cancer remains ambiguous due to the complex cross-talk machinery with apoptosis. Understanding of this mechanism is crucial in developing effective cancer therapy as it is a key regulator in determining the cell fate.

Natural products have attracted the attention of researchers due to their anti-cancer property. 1’S-1-acetoxychavicol acetate (ACA) is a phenylpropanoid isolated from rhizomes of *Alpinia conchigera* Griff. Our group had previously reported the anti-cancer effects of ACA in breast (MCF-7), oral (HSC-2 and HSC-4), liver (HepG2), cervical (CaSki), lung cancer (A549) and prostate carcinoma (PC-3) via inducing apoptosis with minimal cytotoxic effect on normal human mammary cells (HMEC) and no physiological alteration in *in vivo* model [12–14]. It was reported that ACA targets NF-κB signalling pathway to alter the pro-inflammatory microenvironment environment both *in vitro* and *in vivo* [12, 14]. Despite numerous reports on its direct interaction on signalling pathway, ACA can modulate epigenetic machinery in cancer by altering miRNA expression that eventually has an impact in the gene expression [15]. Moreover, a synergistic anti-cancer effect was further observed in combination treatment of ACA with cisplastin or recombinant human alpha fetoprotein [12, 14, 15]. These studies revealed ACA as a potential anti-cancer remedy. Although it was known that ACA induced cytotoxicity against wide range of cancer types, its effect on autophagy remains ambiguous.

The aims of this study were to investigate the autophagy-inducing ability of ACA in human NSCLC. Effect of ACA on cytotoxicity and apoptosis induced was assessed after administering autophagy inhibitors and small interfering RNA (siRNA). Role of ACA-induced autophagy as pro-survival or pro-death mechanism was determined. In present study, we have shown that ACA triggered autophagy through non-canonical pathway in NSCLC and autophagy inhibitors can potentiate the cytotoxicity of ACA through apoptosis. Our results suggested autophagy inhibitors as a potential strategy to improve the efficiency of the ACA in future.

## Materials and methods

### Plant material

Rhizomes of wild *Alpinia conchigera* Griff were collected from Jeli (5°42′N 101°50′E) province of Kelantan, east-coast of Penisular Malaysia. No specific permission was required for collecting the sample from this site as they are wildly grown and thi field study did not involve endangered or protected species. The sample was identified by Prof. Dr. Halijah Ibrahim from Institute of Biological Science, University of Malaya. A voucher specimen (KL5049) was deposited in Herbarium of Chemistry Department, Faculty of Sciece, University of Malaya.

### Chemicals and antibodies

ACA was dissolved in dimethyl sulfoxide (DMSO; Fisher Scientific, USA) to serve as a stock solution. The autophagy inhibitors used were 3-methyladenine (3-MA; Calbiochem, USA) and chloroquine diphosphate (CQ; Sigma, USA). The 3-MA was dissolved directly in culture media and the stock solution CQ was prepared in distilled water. Acridine orange solution was obtained from Sigma. Roswell Park Memorial Institute-1640 (RPMI-1640) medium was purchased from GE Healthcare HyClone (USA) whereas alpha-Minimum Essential Medium (α-MEM) was obtained from Nacalai Tesque (Japan). Fetal bovine serum (FBS) was from Sigma (USA).

Primary antibodies included rabbit antibodies against microtubule associated protein 1 light chain 3-I/II (LC3-I/II), p62, GAPDH and mouse antibodies against Beclin-1 were used. Anti-rabbit IgG and anti-mouse IgG horseradish peroxidase (HRP)-conjugated secondary antibody were used. All the antibodies were purchased from Cell Signaling Technology (USA).

### Cell culture

Two human NSCLC cells lines A549 was purchased from America Type Culture Collection (ATCC, USA) and SK-LU-1 was purchased from AseaCycte Sdn. Bhd., Malaysia. A549 cells were cultured in RPMI-1640 medium while SK-LU-1 cells were cultured in α-MEM medium. The media were supplemented with 10% (v/v) FBS and cells maintained at 37°C incubator with 5% CO_2_. A non-tumourigenic human mammary epithelial cells, MCF 10A was purchased from ATCC and cultured in Mammary Epithelial Cell Growth Medium (MEGM) supplemented with 100 ng/ml cholera toxin.

### MTT cell viability assay

Briefly, cells were seeded at a density of 1×10^4^ cells/well in 96-well plate and treated with ACA (0–30 μM) for 24 h. Following incubation, MTT (5 mg/ml) (Merck, Germany) with a volume of 10 μl was added to each well and the cells were incubated at 37°C incubator for 1 h. The formazan crystal was dissolved in 200 μl of DMSO. The absorbance reading at 560 nm with background subtraction at 630 nm was measured by Tecan Sunrise microtitre plate reader using Magellan Software Version 6.3. The IC_50_ of ACA for each of the cell lines was then determined and this IC_50_ was used for the downstream experiments in this study. MCF 10A was used to determine the toxicity of ACA on non-tumourigenic cells. To investigate the time-dependent effect, the cell viability of A549 and SK-LU-1 cells at IC_50_ value was assessed from 0–24 h. To evaluate the effect of autophagy on ACA-induced cell death, the cells were pre-treated with si*LC3* (20 nM) or CQ (20 μM) for 4 h, followed by addition of IC_50_ concentration of ACA for 24 h and the cell viability was measured.

### Morphological observation

Cells were seeded and treated with ACA, 3-MA, CQ standalone or in combination of ACA with inhibitors. At 24 h, the morphology of cells was examined under a bright field Nikon Eclipse TS100 inverted fluorescent microscope (Nikon, Japan) at 200 × magnification.

### Acidic Vesicular Organelles (AVO) staining

Cells were plated in 6-well plate and incubated overnight at 37°C incubator with 5% CO_2_ for adherence. The following day, cells were incubated with ACA for 0, 3, 6, 12 and 24 h or in presence of si*LC3* or CQ for 24 h. At indicated time point, cells incubated with acridine orange (AO) (1μg/ml) (Sigma-Aldrich, USA) at 37°C for 15 minutes. The cells were observed under Nikon Eclipse TS100 inverted fluorescent microscope (Nikon, Japan) using a blue filter (B-2A) at 400 × magnification. The red relative fluorescence intensity (RFI) which represent the AVO was measured using Nikon NIS-BR Element software.

### Analysis of GFP-LC3-II

Cells were seeded at in 6-well plate and incubate overnight in 37°C incubator with 5% CO_2_ for attachment. All cells were transduced with RFP-GFP-LC3-II reagent using commercially available Premo Autophagy Tandem Sensor RFP-GFP-LC3-II Kit (Life technologies, USA) and treated with ACA for 0, 3 and 6 h or in presence of si*LC3* or CQ for 6 h. At indicated time point, the cells were visualized under Nikon Eclipse TS100 inverted fluorescent microscope (Nikon, Japan) using a blue filter at 400 × magnification to detect the neutral pH autophagosome with GFP emission. Cells with five GFP-LC3-II dots were regarded as positive cells. Quantitation of GFP-LC3-II was examined by counting the number of cells with positive punctate dots in a total of 200 GFP-LC3-II cells.

### Western blot analysis

Cellular proteins were extracted using NE-PER® Nuclear & Cytoplasmic Extraction Kit (Pierce, USA) according to manufacturer’s protocol. The lysate proteins were quantitated using bicinchoninic acid (BCA) protein assay kit (Thermo, USA) according to manufacturer’s instruction. For western blot analysis, 20 μg of lysate proteins were resolved using 12% sodium dodecyl sulphate-polyacrylamide gels (SDS-PAGE) and transferred to a nitrocellulose membrane. The membrane was incubated in relevant primary antibody overnight at 4°C at appropriate dilution. Anti-GAPDH antibody was used at a 1:10000 dilution whereas anti-LC3-I/II, anti-p62, and anti-Beclin-1 were used at a 1:1000 dilution. On the following day, membranes were incubated with anti-biotin and HRP-conjugated secondary antibody at a 1:1:1000 dilution at room temperature with agitation. Detection of bound antibody was done by subjecting the membrane to 1: 1 of Western Bright Quantum components (Advansta, USA) for 2 min in the dark. Protein bands were visualized using a chemiluminescent imaging system (Fusion FX7). GAPDH was used for normalization of band intensity by using ImageJ v1.48 (NIH, USA) densitometry software.

### RNA interference

The cells were seeded 24 h before transfection. Small interfering RNA (siRNA) against *LC3* (si*LC3*) (Origene, USA) or universal scrambled negative control siRNA (OriGene, USA) were transfected at a concentration of 20 nM using Dharmfect I tranfection reagent (Dharmacon, USA) according to the manufacturer’s manual. The knockdown efficiency of 3 unique siRNA duplexes was examined using western blot analysis and the one with best knockdown efficiency was selected for the subsequent experiments. Sequence of the 3 unique siRNA duplexes is shown in Table 1.

**Table 1:** Sequence of three unique 27 mer siRNA duplexes targeting *LC3* (OriGene, USA).

siRNA	Duplex sequence
**A**	5’-CCUGUAUACGUUAGUGAAAGCUGTT-3’
**B**	5’-UACAGAUACUAAUGUCAAGAGUUAA-3’
**C**	5’-CGUUUAGACUGUAUACAUCAUAUCT-3’

### Annexin V-FITC/PI apoptosis assay

Apoptosis was measured by annexin V-FITC apoptosis detection kit (Calbiochem, USA) according to the manufacturer’s instruction. Briefly, the seeded cells were pre-treated with si*LC3* (20 nM) or CQ (20 μM) (Sigma-Aldrich, USA) for 4 h, followed by addition of IC_50_ concentration of ACA for 24 h. At indicated time point, both floating and attached cells were harvested, washed with cold PBS, and followed by incubation with 500 μl binding buffer containing 1.25 μl of annexin V-FITC and 10 μl of propidium iodide (PI) in the dark. The samples were analysed using BD FACSCantoII flow cytometer (BD Biosciences, USA) with BD FACSDiva (BD Biosciences, USA) software.

### Statistical analysis

The data are presented as mean ± standard deviation (SD) of at least three independent experiments. The statistical data analysis was performed using one-way ANOVA, followed by *post-hoc* Turkey analysis, with a *p* value of < 0.05 indicating a statistically significant difference.

## Results

### ACA inhibited the cell viability in NSCLC cell lines

**Fig 1:**
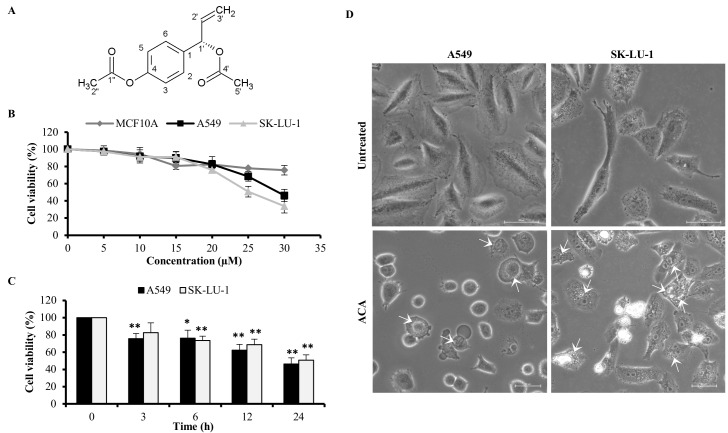
ACA inhibited cell viability of A549 and SK-LU-1 cell lines. (A) Chemical structure of 1’S-1’-acetoxychavicol acetate (ACA). (B) The cell viability of MCF 10A, A549 and SK-LU-1 cells lines after exposure to ACA (0–30 μM) for 24 h was assessed using MTT assay. Data represented as mean percentage of cell viability ± SD for three independent experiments. (C) The cell viability of A549 and SK-LU-1 cells lines after exposure to IC_50_ of ACA (25 μM for SK-LU-1 and 30 μM for A549 cells) on respective cell lines for 0–24 h was assessed using MTT assay. Data represented as mean percentage of cell viability ± SD for three independent experiments. * *p* < 0.05 and ** *p* < 0.01 statistically different in comparison to 0 h (D) Representative photomicrograph (200 × magnification) of A549 and SK-LU-1 cell lines upon ACA treatment. Arrow indicates the cytoplasmic vacuole.

We have previously characterized the structure of ACA (Fig 1A) and reported on its cytotoxicity towards various cancer cell lines [13]. In current study, the cytotoxic effect of ACA treatment in NSCLC cell lines (A549 and SK-LU-1) was investigated by exposing the cells to increasing doses of ACA (0–30 μM) for 24 h. Results indicated ACA elicited a dose-dependent cytotoxicity in both A549 and SK-LU-1 cell lines. The IC_50_ value of ACA in A549 and SK-LU-1 cells were 29.2 ± 1.4 μM and 25.0 ± 1.0 μM respectively. This implied ACA is slightly more sensitive towards SK-LU-1 in comparison to A549 cell lines (Fig 1B). Based on the results from MTT assay, the IC_50_ values of respective cell lines at 24 h were used for subsequent experiments. On the other hand, the cell viability of MCF 10A maintains above 75% after treated with ACA at 30 μM for 24 h with undetermined IC_50_. We also conducted a time-dependent study (0–24 h) of ACA at IC_50_ value in A549 and SK-LU-1 cell line. In Fig 1C, the cell viability of A549 declined more rapidly in comparison to SK-LU-1 at their respective IC_50_ value for ACA (Fig 1C). Under microscopic examination, ACA induced morphological changes in cells as compared to the untreated group with increased cytoplasmic vacuoles in both NSCLC cell lines (Fig 1D); suggesting the possibility of autophagy induction.

### ACA induced autophagy in NSCLC cells

**Fig 2:**
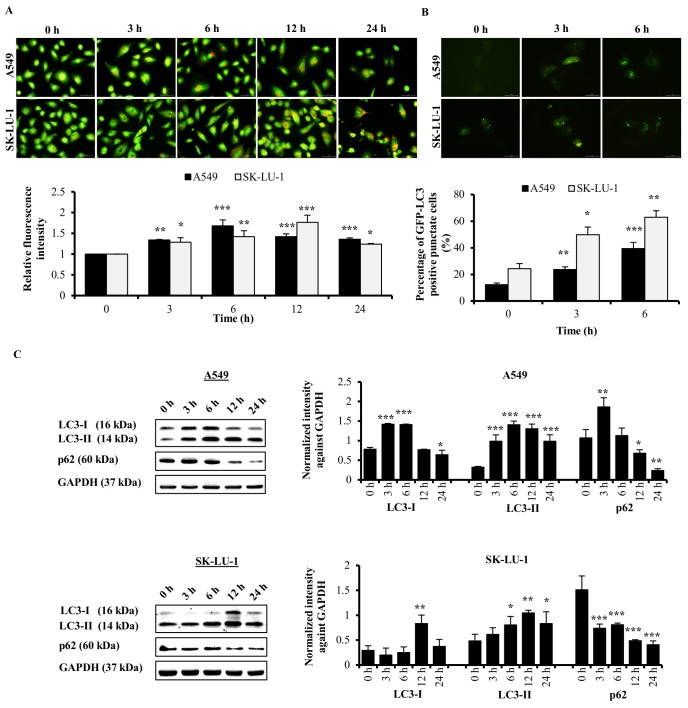
Autophagy effect of ACA on A549 and SK-LU-1 cell lines. (A) Representative fluorescence photomicrograph (400 × magnification) illustrating the acidic vesicular organelles in A549 and SK-LU-1 cell lines after treated with ACA for 0, 3, 6, 12, and 24 h. Data were presented as relative fluorescence intensity in comparison to untreated cells ± SD. * *p* < 0.05, ** *p* < 0.01, and *** *p* < 0.001 statistically different in comparison to untreated. (B) Representative fluorescence photomicrograph (400 × magnification) illustrating the GFP-LC3-II punctate formation in A549 and SK-LU-1 cell lines upon exposure to ACA. Data were represented as mean percentage of cells with GFP-LC3-II punctate ± SD of three independent experiments. * *p* < 0.05, ** *p* < 0.01, and *** *p* < 0.001 statistically different in comparison to untreated. (C) Protein expression of LC3-I/II and p62 after ACA treatment in A549 and SK-LU-1 cell lines. Data were represented as mean normalized intensity ± SD of three independent experiments. * *p* < 0.05, ** *p* < 0.01, and *** *p* < 0.001 statistically different in comparison to untreated.

To investigate the induction of autophagy, AO staining was used to detect the presence of acidic vesicular organelles (AVO). AO stain will emit red fluorescence in acidic vesicles organelles whereas green fluorescence in non-acidic compartment such as cytoplasm and nucleus [16]. After ACA treatment, both AO stained A549 and SK-LU-1 cells exhibited a significant increase in red fluorescence signal that indicated the presence of AVO in comparison to untreated cells (Fig 2A and S1 Fig).

Autophagy activity of ACA was verified by localizing the LC3, a marker of autophagosome, by observing the formation of GFP-LC3-II punctate in the cells. ACA exerted an accumulation of GFP-LC3 punctate in comparison to the diffused pattern of green fluorescence seen in untreated cells (Fig 2B and S1 Fig). Consistent with the results of GFP-LC3-II punctate formation analysis, elevated LC3-II protein expression in A549 and SK-LU-1 cell lines was observed after ACA treatment (Fig 2C). The LC3-II protein expression upon ACA exposure peaked earlier at 6 h in A549 cells compared to 12 h in SK-LU-1 cells. This may indicate that ACA promotes the recruitment of LC3-II into autophagosomes earlier in A549 cells. In the untreated SK-LU-1 cells, a basal level of GFP-LC3-II punctate was seen. However. this level was significantly increased at 3 h (*p* < 0.05) and 6 h (*p* < 0.01) when treated with ACA.

In order to monitor the autophagy flux, we further assessed the degradation of p62 protein expression. Impaired autophagy will lead to accumulation of p62; whereas reduction in expression of p62 indicates an effective autophagy flux [17]. In this study, we noted ACA down-regulated the protein expression of p62 in A549 and SK-LU-1 cells in a time-dependent manner (Fig 2C); indicating a functional autophagy machinery was activated by ACA in these cell lines.

### ACA-induced autophagy is independent of Beclin-1/PI3K complex in NSCLC cell lines

**Fig 3:**
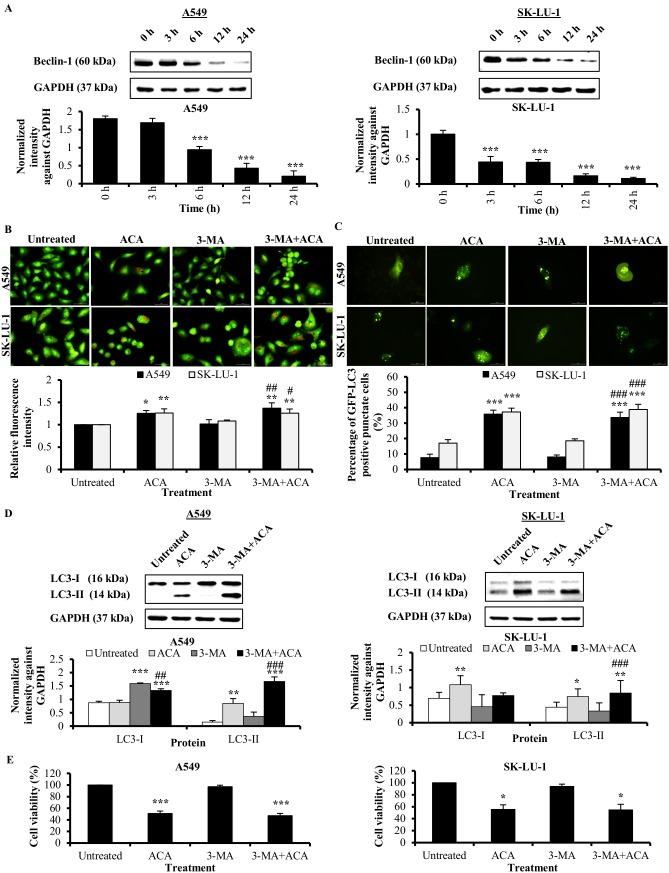
ACA-induced autophagy is independent of Beclin-1/PI3K complex in A549 and SK-LU-1 cell lines. (A) Protein expression of Beclin-1 after ACA treatment in A549 and SK-LU-1 cell lines. Data were represented as mean normalized intensity against GAPDH ± SD. *** *p* < 0.001 statistically different in comparison to untreated. (B) Representative fluorescence photomicrograph (400 × magnification) illustrating the acidic vesicular organelles in A549 and SK-LU-1 cell lines after treatment with ACA in presence or absence of 3-MA. Data were presented as relative fluorescence intensity in comparison to untreated cells ± SD of three independent experiments * *p* < 0.05 and ** *p* < 0.01 statistically different in comparison to untreated. # *p* < 0.05 and ## *p* < 0.01 statistically different in comparison to 3-MA. (C) Representative fluorescence photomicrograph (400 × magnification) illustrating the GFP-LC3-II punctate formation in A549 and SK-LU-1 cell lines upon exposure to co-treatment of 3-MA and ACA. Data were represented as mean percentage of cells with GFP-LC3-II punctate ± SD of three independent experiments. *** *p* < 0.001 statistically different in comparison to untreated. ### *p* < 0.001 statistically different in comparison to 3-MA. (D) Effect of 3-MA on LC3-I/LC3-II protein expression after pre-treatment of 3-MA prior to ACA treatment in A549 and SK-LU-1 cell lines. Data were represented as normalized intensity ± SD of three independent experiments. * *p* < 0.05, ** *p* < 0.01 and *** *p* < 0.001 statistically different in comparison to untreated. ## *p* < 0.01 and ### *p* < 0.001 statistically different in comparison to 3-MA. (E) Effect of 3-MA on the cell viability of ACA-treated A549 and SK-LU-1 cell lines. Data represented as mean percentage of cell viability ± SD for three independent experiments. * *p* < 0.05 and *** *p* < 0.001 statistically different in comparison to untreated.

Studies have shown initiation of autophagy can be Beclin-1-dependent (canonical) or Beclin-1-independent (non-canonical) [7]. To examine the involvement of Beclin-1 in ACA-induced autophagy, we first examined the expression of Beclin-1 protein level using western blot. Our data demonstrated that ACA treatment reduced the expression of Beclin-1 in a time-dependent manner in A549 and SK-LU-1 cells (Fig 3A). Reduction in Beclin-1 protein expression was reported to be representative characteristic of a non-canonical autophagy pathway [18, 19]. Hence, we hypothesized ACA triggered autophagy bypasses the Beclin-1/PI3K complex. In order to verify the non-involvement of Beclin-1/PI3K complex in ACA-induced autophagy, we blocked the Beclin-1/PI3K complex in early process of autophagy using a PI3K class III inhibitor, 3-methyladenine (3-MA). Previous studies showed that 3-MA with concentration of 5 mM can be used to inhibit the early autophagy in lung cancer cells [20, 21]. In these investigations, 3-MA standalone does not show a decrease in basal autophagy and LC3-I expression. Our current study demonstrated administration of 3-MA did not block the red relative fluorescence intensity (RFI) of AO induced by ACA (Fig 3B and S2 Fig), indicating non-involvement of Beclin-1/PI3K complex. Consistently, western blot and GFP-LC3-II revealed that pre-treatment with 3-MA prior to ACA treatment failed to suppress LC3-II protein expression (Fig 3C and 3D and S2 Fig). This further verified that ACA induced Beclin-1/PI3K-independent autophagy. MTT assay was conducted using 3-MA standalone, ACA standalone and combination of 3-MA with ACA to assess the effect of 3-MA on cell viability. The cell viability was maintained at similar level, which is from 55.4 ± 7.9% to 54.7 ± 9.4% and from 50.8 ± 4.2% to 47.0 ± 4.1% in A549 and SK-LU-1cell lines respectively with pre-treatment of 3-MA prior to ACA treatment (Fig 3E). This indicated that 3-MA has no significant effect on cell viability of A549 and SK-LU-1 cells; most probably attributed by the autophagy flux that could not be inhibited using 3-MA.

### Knockdown of *LC3* enhanced ACA-induced cytotoxicity through apoptosis

**Fig 4:**
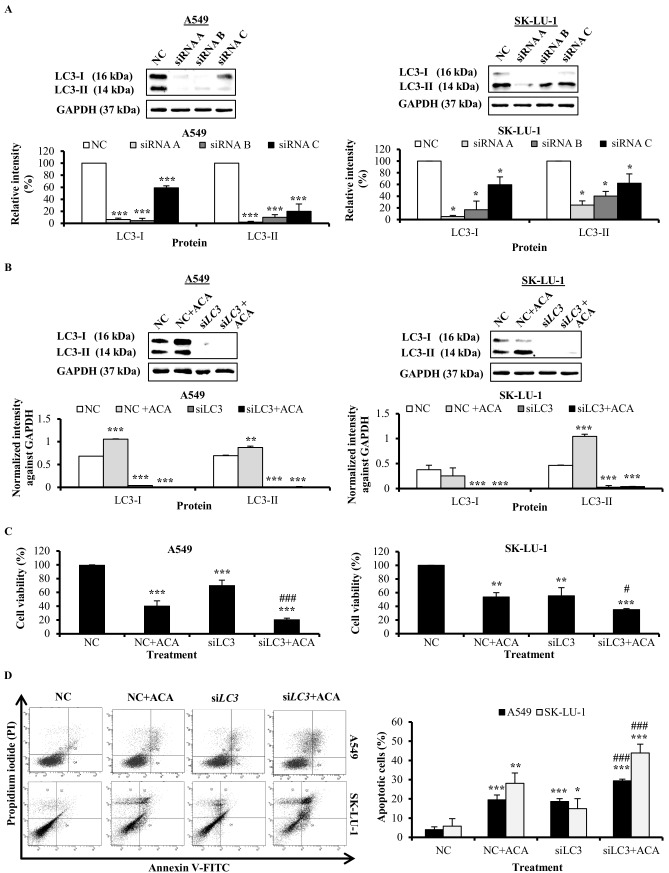
Knockdown of *LC3* enhanced ACA-induced cytotoxicity through apoptosis. (A) Knockdown efficiency of the three unique 27 mer siRNA duplexes targeting *LC3* in A549 and SK-LU-1. Data were represented as mean percentage of relative intensity ± SD of three independent experiments. * *p* < 0.05 and *** *p* < 0.001 statistically different in comparison to negative control (NC). (B) Effect of si*LC3* on ACA-induced LC3-I/LC3-II protein expression in A549 and SK-LU-1 cell lines. Data were represented as normalized intensity ± SD of three independent experiments. *** *p* < 0.001 statistically different in comparison to negative control (NC). (C) Effect of si*LC3* on the cell viability of ACA-treated A549 and SK-LU-1 cell lines. Data represented as mean percentage of cell viability ± SD for three independent experiments. ** *p* < 0.01 and *** *p* < 0.001 statistically different in comparison to negative control (NC). # *p* < 0.05 and ### *p* < 0.001 statistically different in comparison to NC + ACA. (D) Effect of si*LC3* on ACA-induced apoptotic cells in A549 and SK-LU-1 cell lines. Representative annexin V-FITC/PI scatter plots of 1 × 10^4^ cells after 24 h of treatment. Data represented as mean percentage of apoptotic cells ± SD for three independent experiments. * *p* < 0.05, ** *p* < 0.01 and *** *p* < 0.001 statistically different in comparison to negative control (NC). ### *p* < 0.001 statistically different in comparison to NC + ACA.

To clarify the role of ACA-induced Beclin-1/PI3K-independent autophagy in cell death, we inhibited the process of autophagy in NSCLC cells by reducing *LC3* expression, which is the key player in forming autophagsome. First, the efficiency of the 3 unique siRNA duplexes targeting *LC3*, siRNA*3* A, B and C were assessed using western blot. siRNA A targeting *LC* was selected for the downstream experiments due to its best knockdown ability in both A549 and SK-LU-1 cells (Fig 4A). Result showed siRNA A inhibited the protein expression of LC3 successfully in both NSCLC cell lines even when ACA was presented (Fig 4B). After transfected with si*LC3*, the cell viability upon ACA exposure was decreased from 40.8 ± 6.9% to 20.8 ± 1.7% in A549 cells, while 51.0 ± 6.3% to 35.1 ± 1.3% in SK-LU-1 cells (Fig 4C). Administration of ACA increased apoptosis in *LC3* silenced NSCLC cells (Fig 4D). This implies that the autophagy mediated by ACA limits the death inducing effect in these cells and was taking on a pro-survival role.

### Autophagy inhibitor chloroquine enhanced ACA-induced cytotoxicity through apoptosis

**Fig 5:**
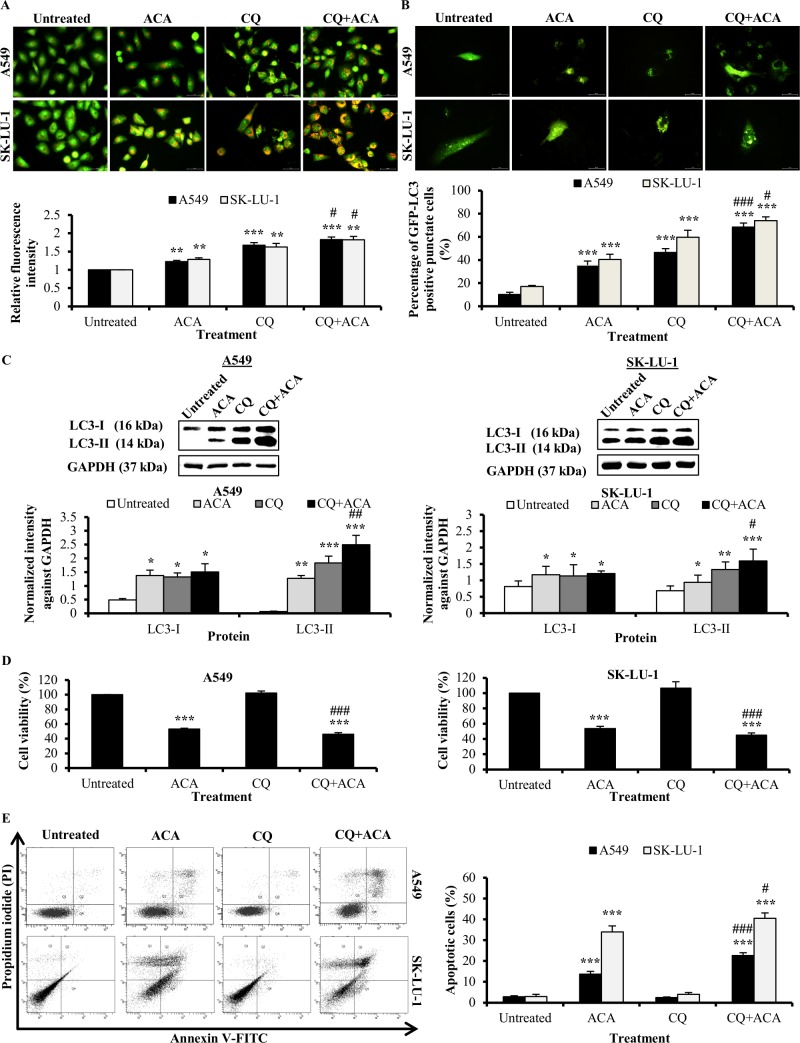
Autophagy inhibitor chloroquine enhanced ACA-induced cytotoxicity through apoptosis. (A) Representative fluorescence photomicrograph (400 × magnification) illustrating the acidic vesicular organelles in A549 and SK-LU-1 cell lines after treatment with ACA in presence or absence of CQ. Data were presented as relative fluorescence intensity in comparison to untreated cells ± SD of three independent experiments. ** *p* < 0.01 and *** *p* < 0.001 statistically different in comparison to untreated. # *p* < 0.05 statistically different in comparison to CQ. (B) Representative fluorescence photomicrograph (400 × magnification) illustrating the GFP-LC3-II punctate formation in A549 and SK-LU-1 cell lines upon exposure to co-treatment of CQ and ACA. Data were represented as mean percentage of cells with GFP-LC3-II punctate ± SD of three independent experiments. *** *p* < 0.001 statistically different in comparison to untreated. # *p* < 0.05 and ### *p* < 0.001 statistically different in comparison to CQ. (C) Effect of CQ on LC3-I/LC3-II protein expression after pre-treated with CQ prior ACA treatment in A549 and SK-LU-1 cell lines. Data were represented as normalized intensity ± SD of three independent experiments. * *p* < 0.05, ** *p* < 0.01 and *** *p* < 0.001 statistically different in comparison to untreated. # *p* < 0.05 and ## *p* < 0.01 statistically different in comparison to CQ. (D) Effect of CQ on the cell viability of ACA-treated A549 and SK-LU-1 cell lines. Data represented as mean percentage of cell viability ± SD for three independent experiments. *** *p* < 0.001 statistically different in comparison to untreated. ### *p* < 0.001 statistically different in comparison to ACA. (E) Effect of CQ on ACA-induced apoptotic cells in A549 and SK-LU-1 cell lines. Representative annexin V-FITC/PI scatter plots of 1 × 10^4^ cells after 24 h of treatment. Data represented as mean percentage of apoptotic cells ± SD for three independent experiments. *** *p* < 0.001 statistically different in comparison to untreated. # *p* < 0.05 and ### *p* < 0.001 statistically different in comparison to ACA.

We further investigated the effect of the inhibitor, chloroquine (CQ) that inhibits lysosomal turnover of autophagosome contents during the ACA-induced autophagy. Administration of CQ (20 μM) alone blocked the fusion of autophagosome and lysosome; leading to accumulation of LC3-II formation and lysosomes. The RFI of ACA in combination with CQ was increased in NSCLC cells; indicating the increase in acidic compartments (Fig 5A and S3 Fig). GFP-LC3-II punctate and western blot showed that CQ further elevated the LC3-II protein level induced by ACA (Fig 5B and 5C and S3 Fig). These data confirmed that ACA stimulated autophagy. In the MTT assay, cell viability was reduced significantly from 53.3 ± 1.0% to 46 ± 2.4% and 53.6 ± 2.9% to 45 ± 2.9% in A549 and SK-LU-1cell lines respectively with pre-treatment of CQ prior to ACA treatment (Fig 5D). In addition, annexin V-FITC/PI staining disclosed that pre-treatment with CQ increased ACA-induced apoptotic cells (Fig 5E). These results proposed the pharmacological inhibition of autophagy using autophagy inhibitor such as CQ can potentiate the cytotoxic ability of ACA to induce apoptosis; thus giving a new perspective for improvement of chemotherapeutic outcome in the future.

## Discussion

The naturally occurring ginger compound, ACA, has been extensively studied which revealed its potential as an anti-cancer agent through activation of apoptosis [12–14]. However, the ability of ACA to induce autophagy in NSCLC remains unclear. Our current study revealed that ACA induced autophagy in NSCLC as evidenced by the accumulation of AVO and GFP-LC3-II along with elevated LC3-II protein level indicating the recruitment LC3-II on autophagosomes. Furthermore, degradation of p62 observed further implied that ACA induced autophagy flux. This is the first study reporting on autophagy inducing ability of ACA in NSCLC. Natural occurring compounds such as curcumin and resveratrol were also found to activate autophagy and/or apoptosis via reactive oxygen species (ROS) pathway in oral and colon carcinoma respectively [22–24]. Previous studies reported that ACA can stimulate the production of ROS in myeloid leukemia and hepatocellular carcinoma [25, 26]. This might be the possible mechanism of ACA regulating autophagy in both A549 and SK-LU-1 cells.

Autophagy is governed by a series of ATG proteins for initiation and followed by formation of autophagosome. One of the major components during formation of autophagosome in canonical autophagy pathway is PI3K complex which consists of Beclin-1, Vps34 and UVRAG/Atg14. Substantial studies demonstrated activation of autophagy can be in a Beclin-1/PI3K-independent manner. For instance, gossypol can initiate autophagy in the absence of Beclin-1 in HeLa cells but dependent on Vps34 and Atg5; verifying Beclin-1-independence [27]. Earlier study also provided evidence on existence of Beclin-1-independent autophagy induced by resveratrol in human breast cancer cells when 3-MA was unable to suppress autophagy [28]. It was reported autophagy activated by proteasome inhibitors and arsenic trioxide is concomitant with reduction in Beclin-1 expression in ovarian carcinoma cells; suggesting a Beclin-1-independent pathway [29, 30]. These studies provided evidences on the existence of non-canonical autophagy. In the current study, we found that autophagy induced by ACA was independent of Beclin-1/PI3K complex which was reflected in the down regulation of Beclin-1. Furthermore, 3-MA, a PI3K inhibitor was unable to block ACA-induced autophagy and did not potentiate the cytotoxic effect of ACA. An earlier report has identified NF-κB p65/Rel A as a positive regulator of Beclin-1 expression [31]. Since ACA was found to inhibit p65/Rel A previously [14], it is possible that the reduction of Beclin-1 observed upon ACA treatment in this study was as a result of the low expression of p65/Rel A. However, the underlying mechanism for formation of autophagosome which bypass the Beclin-1/PI3K complex and the specific function of the non-canonical autophagy in cancer remains to be further investigated.

Autophagy is a double-edged sword that plays a significant role in tumourigenesis and cancer therapeutics. Hence, anti-cancer agents that modulate autophagy may have an important implication in clinical application. Anti-cancer agents have been found to induce pro-survival autophagy which eventually contributes to the chemoresistance in NSCLC. Innate resistance to erlotinib in NSCLC with wild-type epidermal growth factor receptor (EGFR) was as a result of autophagy [32]. Likewise, gefitinib which is initially effective for inducing death in NSCLC, was seen to develop resistance due to the pro-survival autophagy [33]. Morover, a recent finding published by Wang *et al*. indicated knock down of *LC3* uing siRNA has a significant impact on the cell viability of cancerous cells; suggesting LC3 plays an important role in controlling the cell death [34]. These implied that targeting genes responsible for autophagy can be a strategy for treating chemoresistance that resulted from protective autophagy. In the current study, we observed that the ACA-induced apoptosis was enhanced in A549 and SK-LU-1 cells when *LC3* was silenced. It reflected that autophagy induced by ACA in NSCLC is limiting the apoptotic cell death.

Studies have shown that gefitinib- and erlotinib-resistance in NSCLC can be resolved by combine therapy with CQ [32, 33]. For instance, Zou and coworkers reported that CQ has no growth inhibitory effect on NSCLC even in prolong erlotinib treatment. It also potentiated the anti-cancer effect of erlotinib [32]. Our present study demonstrated combination of CQ with ACA exhibited a synergistic anti-cancer effect of ACA as shown in the MTT assay and annexin V/PI apoptosis assay. Currently, autophagy inhibitors such as CQ and hydroxychloroquine (HCQ) are under investigation in clinical phase I/II for various cancers including lung cancer [17, 35]. Results from phase I study had provided a strong evidence that HCQ with or without erlotinib does not elicit adverse side effects in advance NSCLC patients [36]. Although available data showed the autophagy inhibitors in combination with anti-cancer agents have been recognised as a potential strategy to improve the efficiency of the available therapeutic agents and avoid acquired resistance; the role of autophagy requires further elucidation in other cancer types due to the fact that the autophagy stimulated by a single compound could have different role in different cancer types at different stage of cancer development [35].

## Conclusion

Collectively, our current study demonstrated for the first time that ACA induced autophagy is through Beclin-1-independent/non-canonical pathway in NSCLC. In addition, we showed autophagy inhibitor CQ or inhibition of autophagy gentically by knockdown of *LC3* using siRNA promoted ACA-induced cell death through the apoptosis mechanism. These knowledge suggested targeting autophagy mechanism in cancer therapy may be a promising approach to sensitized NSCLC to ACA treatment.

## Supporting information

S1 Fig Photomicrograph of A549 and SK-LU-1 upon ACA treatment. (A) Representative fluorescence photomicrograph (400 × magnification) illustrating the acidic vesicular organelles in A549 and SK-LU-1 cell lines after treatment with ACA. Arrow indicates the acidic vesicular organelles. (B) Representative fluorescence photomicrograph (400 × magnification) illustrating the GFP-LC3-II punctate formation in A549 and SK-LU-1 cell lines after ACA treatment. Arrow indicates the GFP-LC3-II punctate.
(TIF)

S2 Fig Photomicrograph of A549 and SK-LU-1 after treatment with ACA in presence or absence of 3-MA. (A) Cells were treated with 3-MA in presence or absence of ACA. Arrow indicates the cytoplasmic vacuole. (B) Representative fluorescence photomicrograph (400 × magnification) illustrating the acidic vesicular organelles in A549 and SK-LU-1 cell lines after treatment with ACA in presence or absence of 3-MA. Arrow indicates the acidic vesicular organelles. (C) Representative fluorescence photomicrograph (400 × magnification) illustrating the GFP-LC3-II punctate formation in A549 and SK-LU-1 cell lines upon exposure to co-treatment of 3-MA and ACA. Arrow indicates the GFP-LC3-II punctate.
(TIF)

S3 Fig Photomicrograph of A549 and SK-LU-1 after treatment with ACA in presence or absence of CQ. (A) Cells were treated with CQ in presence or absence of ACA. Arrow indicates the cytoplasmic vacuole. (B) Representative fluorescence photomicrograph (400 × magnification) illustrating the acidic vesicular organelles in A549 and SK-LU-1 cell lines after treatment with ACA in presence or absence of CQ. Arrow indicates the acidic vesicular organelles. (C) Representative fluorescence photomicrograph (400 × magnification) illustrating the GFP-LC3-II punctate formation in A549 and SK-LU-1 cell lines upon exposure to co-treatment of CQ and ACA. Arrow indicates the GFP-LC3-II punctate.
(TIF)
