# Supplementary figures and images for: The apoptotic effect of 1’S-1’-Acetoxychavicol Acetate (ACA) enhanced by inhibition of non-canonical autophagy in human non-small cell lung cancer cells
Source: PLoS One. 2017 Feb 3;12(2):e0171329. doi: 10.1371/journal.pone.0171329 (PMC5291426; doi:10.1371/journal.pone.0171329)

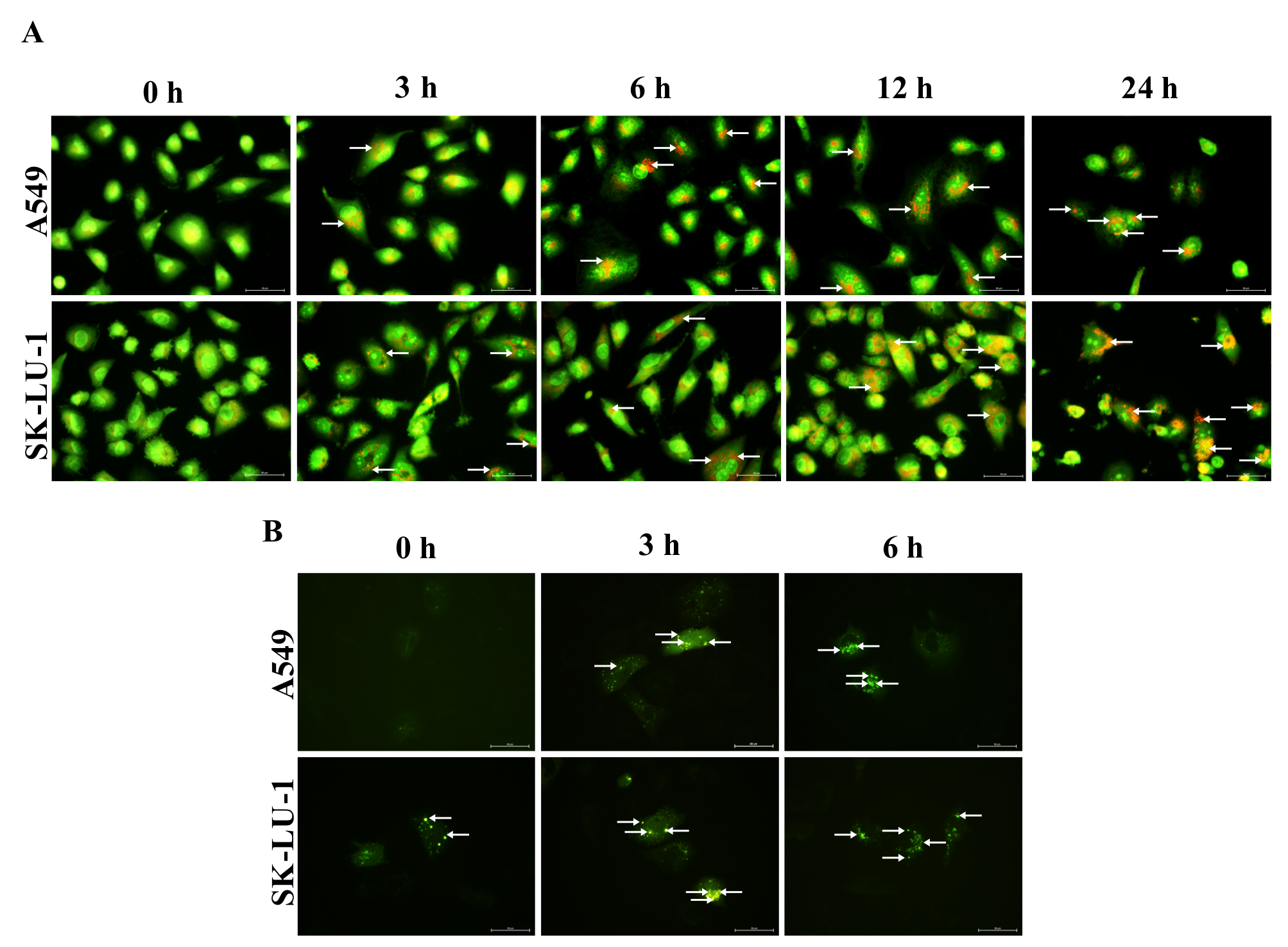

Supplement: S1 Fig — (A) Representative fluorescence photomicrograph (400 × magnification) illustrating the acidic vesicular organelles in A549 and SK-LU-1 cell lines after treatment with ACA. Arrow indicates the acidic vesicular organelles. (B) Representative fluorescence photomicrograph (400 × magnification) illustrating the GFP-LC3-II punctate formation in A549 and SK-LU-1 cell lines after ACA treatment. Arrow indicates the GFP-LC3-II punctate. (TIF) [file pone.0171329.s001.tif]

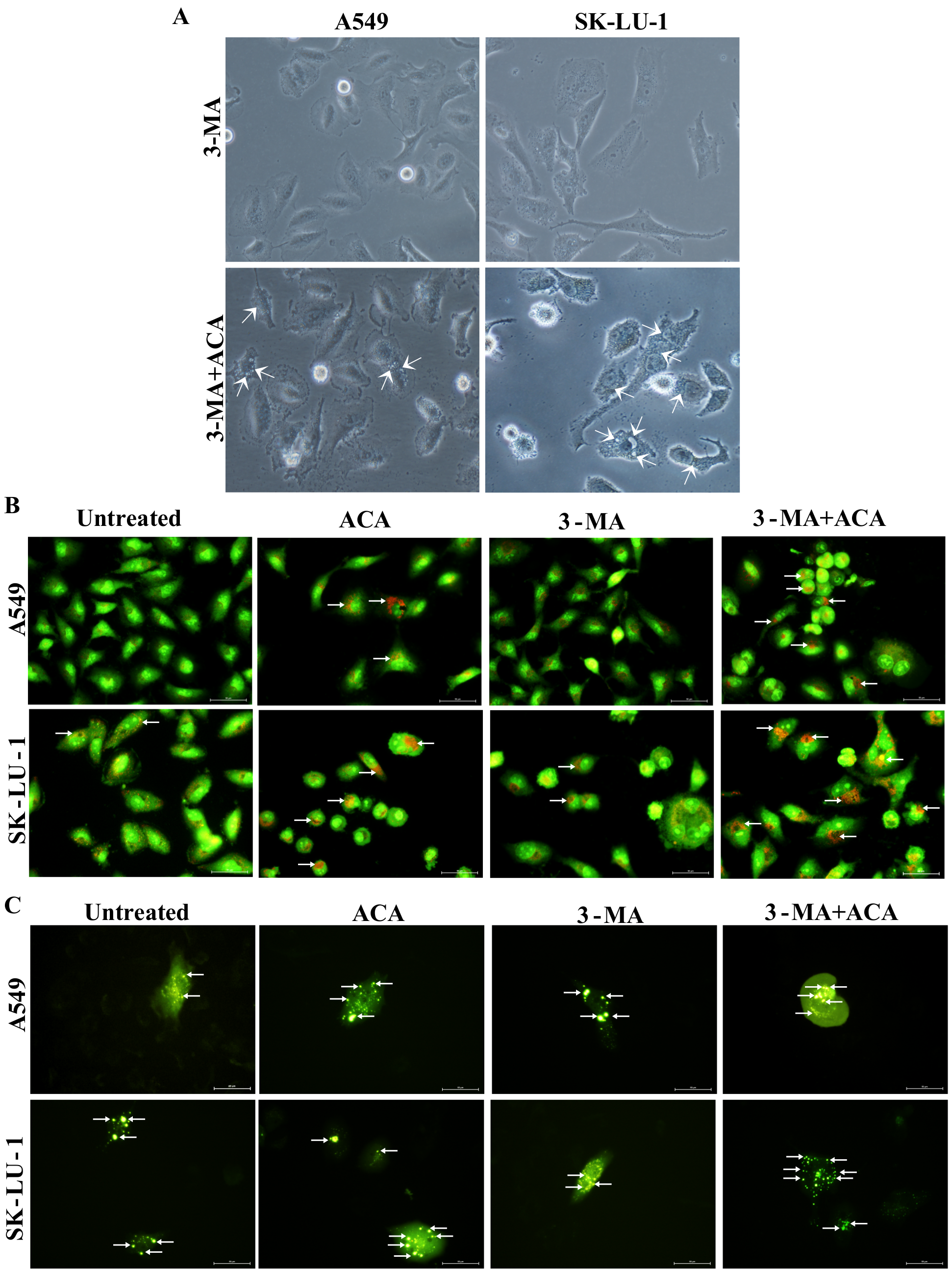

Supplement: S2 Fig — (A) Cells were treated with 3-MA in presence or absence of ACA. Arrow indicates the cytoplasmic vacuole. (B) Representative fluorescence photomicrograph (400 × magnification) illustrating the acidic vesicular organelles in A549 and SK-LU-1 cell lines after treatment with ACA in presence or absence of 3-MA. Arrow indicates the acidic vesicular organelles. (C) Representative fluorescence photomicrograph (400 × magnification) illustrating the GFP-LC3-II punctate formation in A549 and SK-LU-1 cell lines upon exposure to co-treatment of 3-MA and ACA. Arrow indicates the GFP-LC3-II punctate. (TIF) [file pone.0171329.s002.tif]

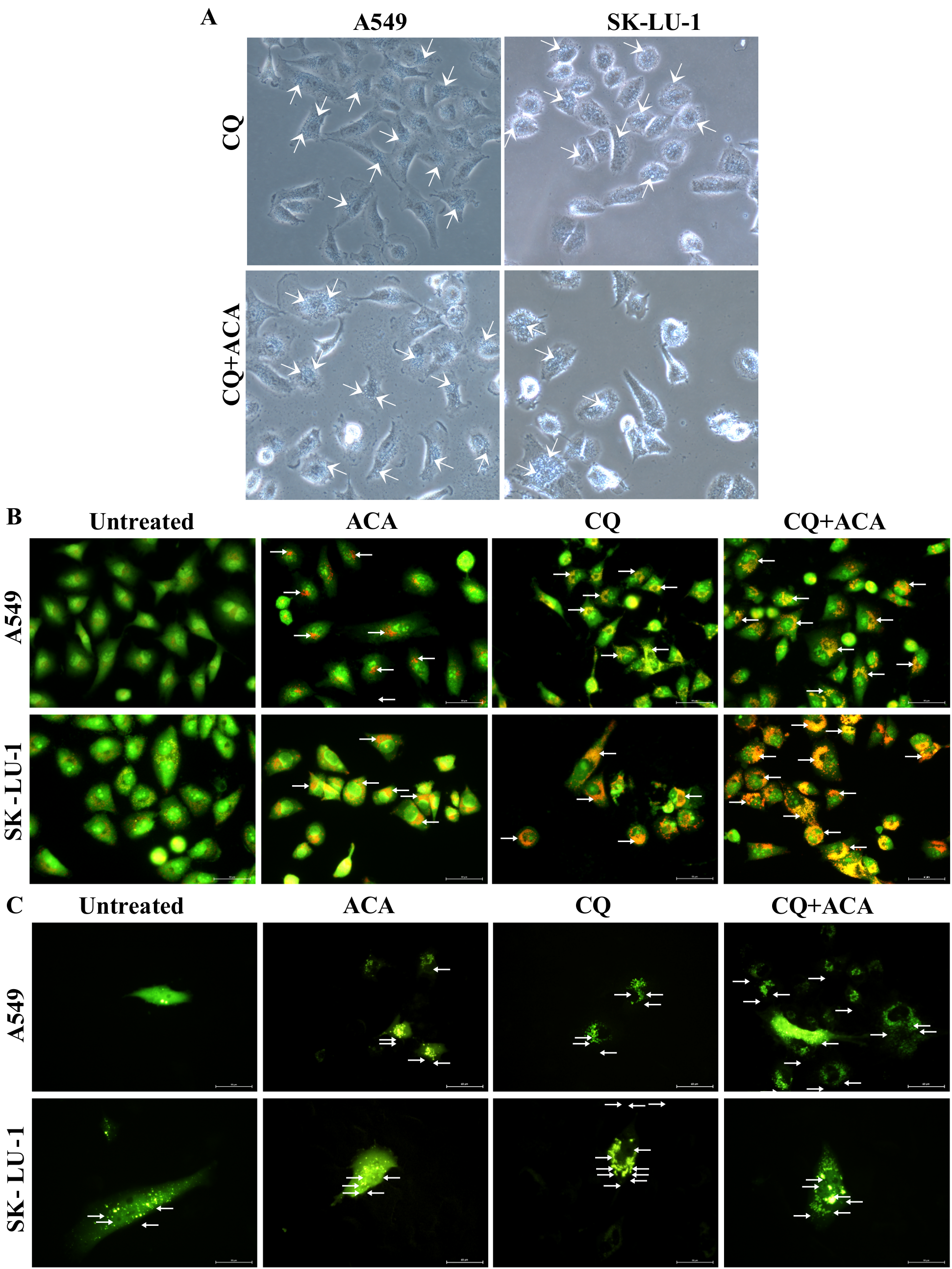

Supplement: S3 Fig — (A) Cells were treated with CQ in presence or absence of ACA. Arrow indicates the cytoplasmic vacuole. (B) Representative fluorescence photomicrograph (400 × magnification) illustrating the acidic vesicular organelles in A549 and SK-LU-1 cell lines after treatment with ACA in presence or absence of CQ. Arrow indicates the acidic vesicular organelles. (C) Representative fluorescence photomicrograph (400 × magnification) illustrating the GFP-LC3-II punctate formation in A549 and SK-LU-1 cell lines upon exposure to co-treatment of CQ and ACA. Arrow indicates the GFP-LC3-II punctate. (TIF) [file pone.0171329.s003.tif]
